# Supplementary material for: Tunnel junctions based on interfacial two dimensional ferroelectrics
Source: Nat Commun. 2024 May 24;15:4449. doi: 10.1038/s41467-024-48634-1 (PMC11126694; doi:10.1038/s41467-024-48634-1)
Supplement: Supplementary file 1 — Supplementary Information [file 41467_2024_48634_MOESM1_ESM.pdf]

Supplementary Information for

Tunnel junctions based on interfacial two dimensional ferroelectrics

Yunze Gao[1,2], Astrid Weston[1,2], Vladimir Enaldiev[1,2], Xiao Li[1,2], Wendong Wang[1,2], James E. Nunn[3], Isaac Soltero[1,2], Eli G Castanon[1,2], Amy Carl[1,2], Hugo De Latour[1,2], Alex Summerfield1[,2], Matthew Hamer[1,2], James Howarth[1,2], Nicholas Clark[1,2], Neil R. Wilson[3], Andrey V. Kretinin[1,2,4*], Vladimir I. Fal'ko[1,2,5,*] and Roman Gorbachev[1,2,5,*]

1 Department of Physics and Astronomy, The University of Manchester, Oxford Road, Manchester, M13 9PL, UK
2 National Graphene Institute, The University of Manchester, Oxford Road, Manchester, M13 9PL, UK
3 Department of Physics, University of Warwick, Coventry, CV4 7AL, UK
4 Department of Materials, The University of Manchester, Oxford Road, Manchester, M13 9PL, UK
5 Henry Royce Institute for Advanced Materials, The University of Manchester, Oxford Road, Manchester, M13 9PL, UK

E-mail: andrey.kretinin@manchester.ac.uk, vladimir.falko@manchester.ac.uk, roman@manchester.ac.uk.
First three authors contributed equally.

1. Rhombohedral (R)-stack twisted bilayer MoS_2 FTJ Sample Fabrication

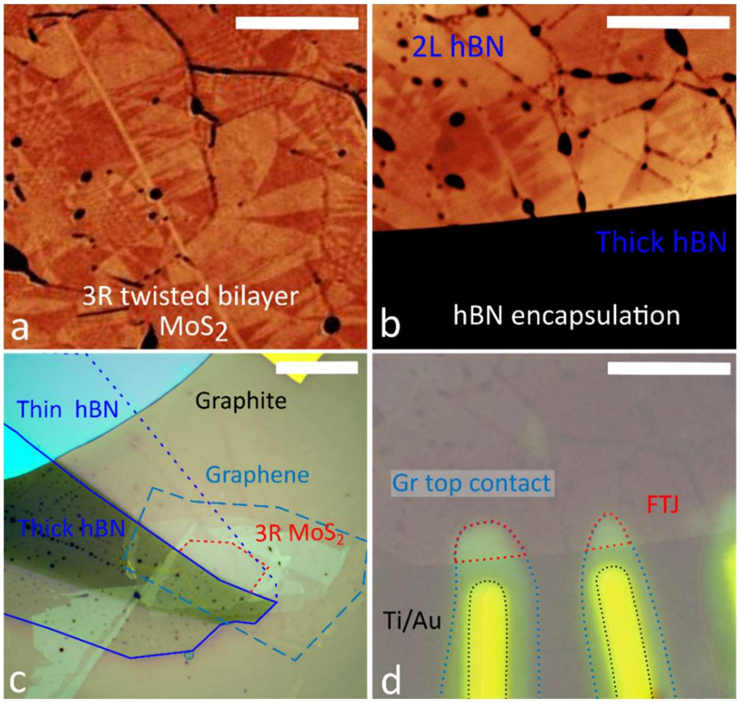

Supplementary Figure S1 Fabrication process of 3R twisted bilayer MoS_2 FTJs (a) Tapping mode topography image of a characteristic twisted bilayer MoS_2/graphite sample. (b) Tapping mode topography image of MoS2/graphite stack after encapsulation with a bulk hBN crystal with a region of few layer hBN attached. (c) optical micrograph image of the completed vdWs heterostructure prior to RIE etching and Ti/Au electrode deposition (scale bar 20 um). (d) Optical micrograph image of the completed sample overlayed onto the tapping mode topography image seen in (b) to indicate the area of individual the tunnelling junctions (scale bars for (a-b) and (d) are 2 um.

Twisted R-stacked MoS_2 bilayers were fabricated using a polymer-assisted (poly-methyl methacrylate (PMMA)) dry transfer technique, typically used for building vdW heterostructures. We employed a modified tear-and-stack technique [1] as previously used in our earlier work [2] using a remotely controlled micromanipulation transfer rig housed inside an argon atmosphere. Monolayers of MoS_2 were mechanically exfoliated onto Si/SiO_2 (90nm) coated with polypropylene carbonate (PPC). A poly-methyl methacrylate (PMMA) carrier layer was used to pick up one half of a MoS_2 flake and subsequently the second half with a rotation angle of $\theta \approx 0^o$. The suspended twisted bilayer was transferred onto a graphite crystal exfoliated onto a Si/SiO_2 (90 nm) substrate. The sample was then annealed at 200 C in vacuum to relax the domain structure and prevent its future movement. The domain structure of the exposed MoS_2 was mapped (see supplementary Fig. S1a) using contact mode and/or tapping mode AFM (Bruker FastScan). Following this, a bulk hBN crystal with attached few-layer hBN was transferred over the region of interest. The sample was annealed again at 180 C in vacuum, enabling routing of the FTJ contacts. The domain structure mapping procedure was then repeated to check whether there were any changes (if any) to the underlying domain structure during

the transfer process (as seen in supplementary Fig. S1b). It is essential to fully characterise the domain structure prior to transferring the top graphene contact because the graphene itself causes electrostatic screening of the domain contrast signal. Finally, a top graphene electrode was transferred onto the vdW heterostructure followed by the final annealing step at 180 C in vacuum.

Electron-beam lithography was used to pattern an etch mask in a 2-layer PMMA resist (3% 495 K followed by 3% 950K PMMA) for reactive ion etching (RIE) to shape the individual top graphene contacts. To create individual FTJ devices at specified areas RIE was performed in a vacuum chamber (pressure 2 x10^{-2} mbar) with a flowing mixture of argon and oxygen with a ratio of 2:1 with a forward power of 10 W to selectively etch the top graphene contact (10-30 sec etch time, depending on sample). Electron-beam lithography and subsequent lift-off was then used to pattern Ti/Au wires contacting the top graphene and bottom graphite. To achieve optimum metal deposition, a beam voltage of 9.5 kV and a beam current of 30-35 mA was used for titanium and gold deposition on a Moorfield E-beam evaporation system. This achieved an average deposition rate of 0.1 Å/s and 1.1 Å/s, respectively.

2. Band alignment

While extensive literature on the band alignment between TMDs, hBN and graphene exists [34567], variation due to doping of hBN and MoS_2 makes this value dependent on the specific growth technique used. To avoid ambiguity on the values used in this work, we have conducted ARPES measurements of individual pairs of materials exfoliated from the same source materials used in the tunneling devices reported in the main text.

We first focus on the band alignment of exfoliated graphene placed on multilayer hBN substrate. Both K point of graphene and hBN can be clearly seen in the ARPES scans, Fig. S2. We extract the offset between the valence band edge of hBN and the Dirac point of graphene of 2.66 eV. Using the band gap value of hBN of 5.95[8,9] we get the band alignment between the conduction band edge of hBN and the Dirac point in graphene, U_{hBN} of 3.29 eV.

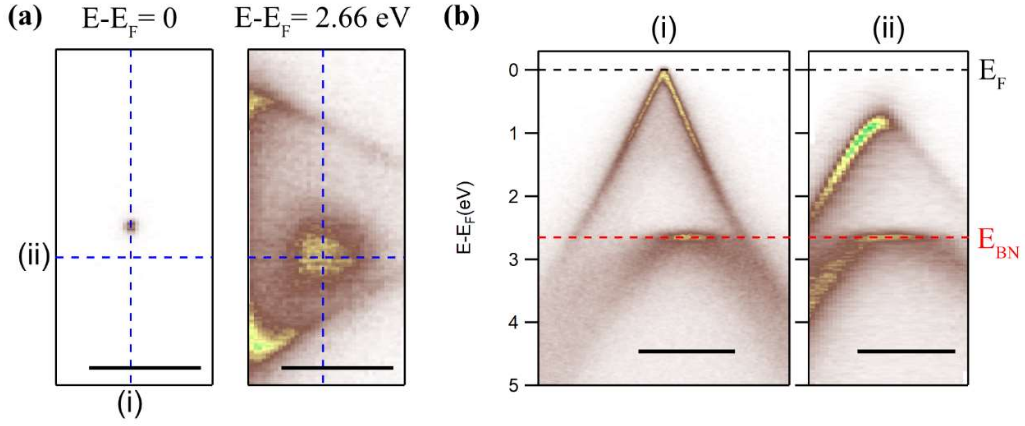

Supplementary figure *S2:* ***ARPES maps of graphene on bulk hBN.*** **(a)** Shows constant energy slice through the graphene's Dirac point (left) and the valence band edge of hBN (right). Slices along the blue dashed lines shown in **(b).**

Similarly, we study band alignment between graphene and MoS_2 bilayer. Although graphite drain is used in the FTJ devices, it is not possible to visualise graphite and MoS_2 bilayer in ARPES as it is essentially a surface technique and placing graphite over MoS_2 will result in absence of signal from the MoS_2. For that reason, we use graphene on 2L MoS_2, and observe graphene Dirac point binding energy of E_D= -0.3 +/- 0.05 eV, while VBM of the MoS_2 at Gamma has the binding energy of E_{VBM}=1.12+/-0.05 eV. Using the single particle gap value of 1.6 eV for bilayer MoS_2[10], we estimate the energy difference between graphene Dirac point and the MoS_2 conduction band edge as U_{MoS2} = 0.2 eV. As we anticipate the difference between graphene and graphite substrates, we use the more relevant value of U_{MoS2} = 0.3 eV from [11].

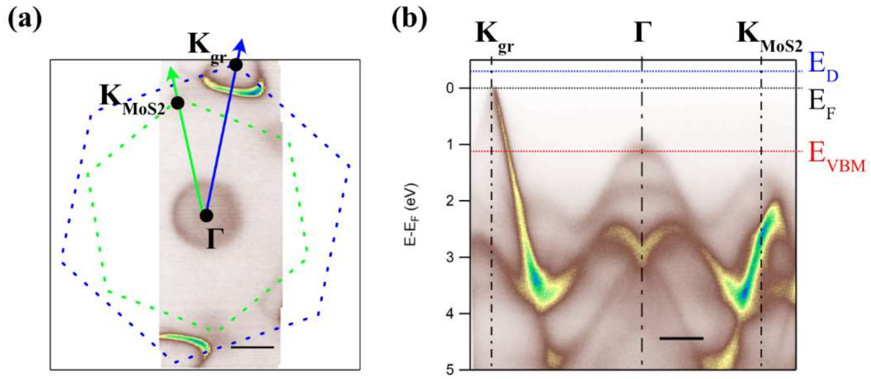

Supplementary figure *S3:* ***ARPES maps of graphene on 2L MoS2.*** **(a)** Shows constant energy slice through the valence band edge of MoS2 bilayer. The slice along the blue solid lines shown in **(b)** used for the extraction of the band alignment **.**

AFM imaging technique for domain mapping

In order to spatially map the domain structure of the R-stacked twisted bilayer MoS2 in our FTJ devices, a combination of contact mode (lateral or piezo-response) and tapping mode was used. All AFM imaging was performed on a Bruker Dimension Icon AFM. All Imaging was acquired using Budget Sensors ElectriMulti75-G probe with a nominal force constant of 3 N m^{-1}, a nominal resonant frequency of 75 kHz and a conductive coating of 5 nm Cr and 25 nm Pt. The conductive coating significantly enhanced the domain contrast in all the modes utilized, likely due to the electrostatic forces between the sample and the conductive probe. The selection of AFM mode used was dependent on the cleanliness of the MoS_2 surface and the quality of the domain contrast. For example, contact mode AFM was selected for samples with significantly higher surface contamination (hydrocarbons and polymer residue) whereby scanning in contact with the surface provided a means to remove the surface hydrocarbons. During contact mode imaging, the friction channel was used to map the domains (see Fig. 1 in the main text). Typical imaging force set points were 30-40 nN.

For (single frequency) PFM imaging, the contact resonant frequency (typically 300-350 kHz) was used with a typical AC voltage of 500 mV applied to the probe.

For the tapping mode imaging, the oscillation setpoint was optimized such that the acquired images were in the attractive regime [12] in order to reveal the domain structure.

In the lateral mode, the images were acquired with a typical force setpoint of 30-40 nN when driven at 5% below the resonant frequency. The topography channel was used to map the domain structure (see supplementary Fig. 1a and b). All AFM data analysis was performed using the Gwyddion scanning probe data analysis software [13].

3. FTJ electrical transport measurement technique

As a semiconductor, bilayer MoS_2 has a relatively low bandgap (∼ 1.2eV). Therefore, in a bilayer semiconductor FTJ, the electric field required for switching its polarisation could produce a destructive tunnelling current. In our devices, the MoS_2 bilayer is encapsulated by an ultra-thin hBN layer such that when the polarisation of the FTJ switched, the electric field can only generate measurable and non-destructive tunnelling current (≤1μA). When measuring the tunnelling conductance as a function of the sweeping transverse field, a constant small AC bias (1mV) and a sweeping DC bias were applied to the graphene source with a SR860 lock-in amplifier while the graphite drain was connected to a SR560 pre-amplifier which amplified the tunnelling current into a measurable voltage bias with

respect to the virtual ground in the pre-amplifier. The tunnelling conductance is defined as the ratio of the AC component of the tunnelling current and the AC bias. The tunnelling current is too small to be measured until the DC bias increases to about 250mV, as a result the small AC bias can be assumed as a negligible fluctuation to the source and drain potential. The whole measurement was performed at 1.5K.

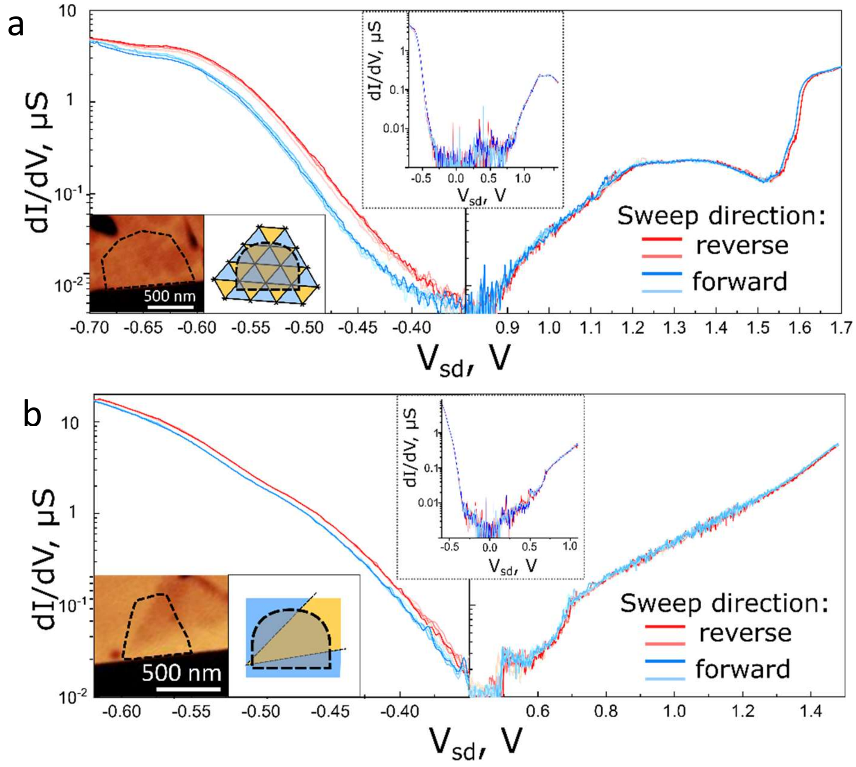

Supplementary Figure S4 Polarisation switching in FTJ with different domain layout and various nucleation scenarios. Tunnelling conductance (dI/dV) as a function of the transverse electric field (V_{sd}) between the graphene source and the graphite drain for: (a) periodic triangular domain network (L~ 60-150nm) (b) three domains with domain walls pinned at the edges of tunnelling area using RIE. Tunnelling hBN thickness is 2 layers. This hysteresis observable for (a) and (b), reaching 17 mV in (a) and 7mV in (b) for the negative Vsd side. For the positive side due to the asymmetry of the junction no measurable current is observed up to 0.9V, by which point the domain switching has occurred and no measurable hysteresis is seen above this value.

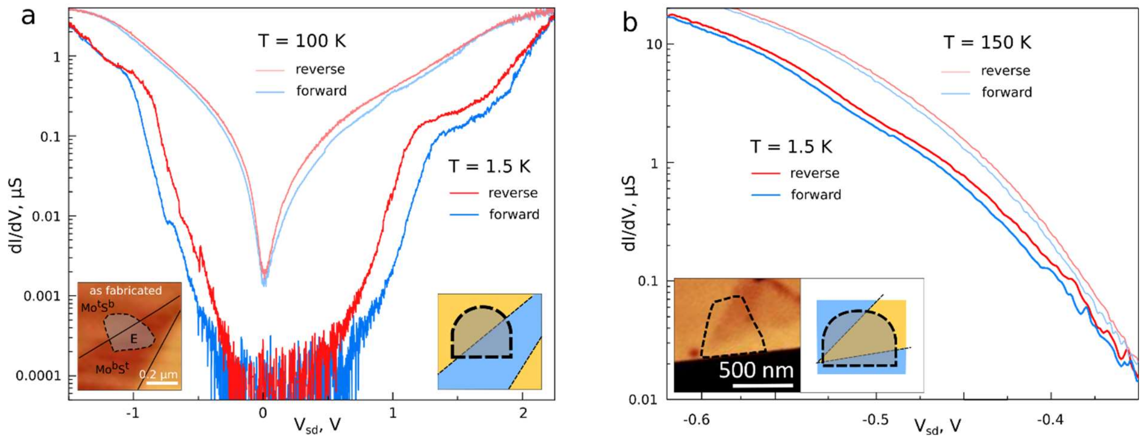

Supplementary *F*igure S5 Switching behaviour at different temperatures. Tunnelling conductance (dI/dV) as a function of the transverse electric field (V_{sd}) between the graphene source and the graphite drain. The direction of the V_{sd} sweeps is indicated by the colour. Schematic insets show the domain configuration that produces the observed tunnelling behaviour, where the area of the FTJ electrode is indicated in grey. The bottom-left inset shows a friction AFM map with the pre-existing domain configuration. (a) is for the device presented in Fig.2 of the main text and (**b**) for the device in Fig.3b of the main text.

The switching behaviour can also be observed at higher temperatures, see Supplementary Figure S5. We observe significant increase in the tunnelling currents which is not consistent with normal thermal contribution to the tunnelling process, and most likely is due to the thermal excitation of electrons from the graphite drain electrode into the conduction band of MoS_2. This produces screening of the ferroelectric potential which leads to decrease in the hysteresis (Eq. 2 main text).

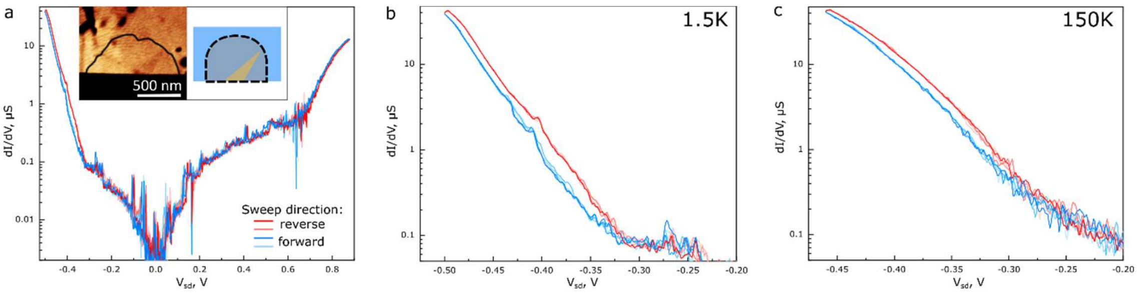

Supplementary *F*igure S6 Switching behaviour in additional device at different temperatures. (**a**) Tunnelling conductance (dI/dV) as a function of the transverse electric field (V_{sd}) between the graphene source and the graphite drain. The direction of the V_{sd} sweeps is indicated by the colour. Schematic insets show the domain configuration that produces the observed tunnelling behaviour, where the area of the FTJ electrode is indicated in grey. The top-left inset shows a friction AFM map with the pre-existing domain configuration similar to that in Fig.3b of the main text. The hBN barrier thickness for this device is 1L and therefore a substantially higher tunnelling current can be observed in the low bias range. (**b, c**) the negative bias range of the curve shown in (a) where the pronounced hysteresis is observed, measured at 1.5K (b) and 150K (c).

4. Reference 2H FTJ device

To test the device design and characterize its quality (e.g. extracting the carrier concentration of the graphene electrodes as well as using the position of the Dirac point to account for any extrinsic doping) we first tested a non-ferroelectric 2H bilayer reference device. For this, the top graphene electrode had multiple contacts as shown in supplementary Fig. S4a, the Dirac point has an offset of ~ 0.6 V,

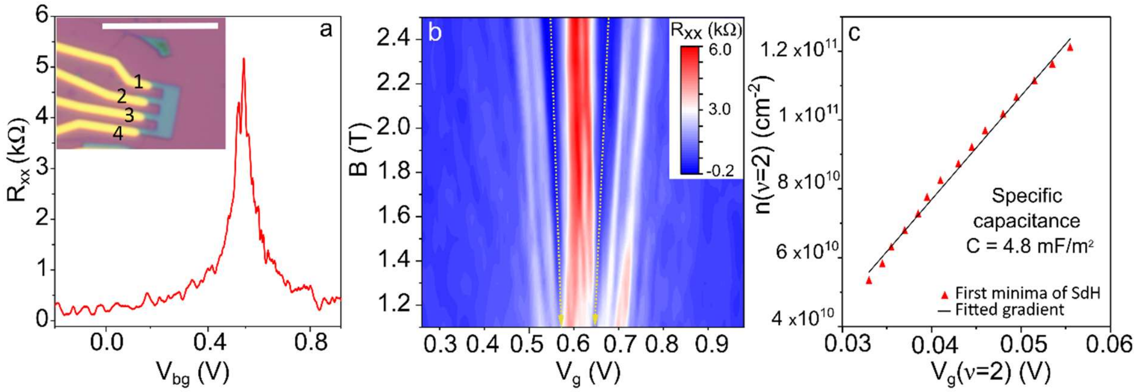

presumably due to surface absorbates.

Supplementary *Figure S7*: Electrical transport measurements of the 2H reference device with Hall bar geometry. (a) Longitudinal resistance R_{XX} as a function of back-gate voltage VBG, where R_{XX} was measured between contacts 2 and 3. Optical image of the 2H-type reference device shown in the inset. Scale bar is 5 um. (b) A colour map of the longitudinal resistance R_{XX} with respect to gate voltage Vg for magnetic fields up to 2.5 T highlighting the presence of Shubnikov-de Haas oscillations. The first minima of R_{XX} are indicated by the dashed yellow lines. (c) The extracted first minima of the Shubnikov-de Haas oscillations, where the gradient is $(n_i/-V_g)e$, was used to calculate specific capacitance C.

We then used the carrier concentration to accurately calculate the capacitance of our 2H bilayer MoS_2/hBN stack. This was achieved by analysing the magnetic field-induced Shubnikov-de Haas (SdH) oscillations at 1.5 K in a four-terminal geometry. Here, we extracted the first minima of the sdH oscillations and plotted it as a function of gate voltage. Here, the gradient $(n_i/-V_g)e$ is equal to the capacitance per unit area (C): $C = -n_i/V_g = \epsilon\epsilon_0/de$, where ∈ (~ 3.6) is the dielectric constant of SiO_2 and d is the thickness of the dielectric (290 nm). From the calculated plot in supplementary Fig. S4c, we obtain a specific capacitance of 4.8 mF/m^2.

5. Theoretical modelling of the ferroelectricity-induced I-V hysteresis

First, we set a model describing electrostatics of the tunnelling device. Applied voltage, $V_{\rm sd}$, drops in graphene and tunneling barriers layers (hBN and MoS_2), which can be described by the following equation:

$$V_{\rm sd} = V_{\rm Gr} - \mathcal{F}_{\rm MoS} d_{\rm MoS} - \mathcal{F}_{\rm BN} d_{\rm BN} \mp \frac{\Delta}{e}. \qquad \text{(S1)}$$

Here, $V_{\rm Gr}$ is the electric potential of graphene related with carrier charge density $Q_{Gr} = \frac{1}{2} C(V_{\rm Gr}) V_{\rm Gr}$ by means of quantum capacitance [T. Fang et al., *Appl. Phys. Lett.* **91**, 092109 (2007)] $C(V_{\rm Gr}) = \frac{2e^3 V_{\rm Gr}}{\pi(\hbar v)^2}$, $\mathcal{F}_{\rm MoS/BN} = -\frac{Q_{Gr}}{\epsilon_0 \epsilon_{\rm MoS/BN}}$ is the electric field across hBN/MoS_2 layers of the structure. By solving (S1) we find

$$V_{\rm Gr}(V_{\rm sd}) = \mathrm{sign}\left(V_{\rm sd} \pm \frac{\Delta}{e}\right)\left[-\frac{V_0}{2} + \sqrt{\left(\frac{V_0}{2}\right)^2 + V_0 \left|V_{\rm sd} \pm \frac{\Delta}{e}\right|}\right], \qquad \text{(S2a)}$$

$$\mathcal{F}_{\rm MoS} = -\frac{\left(V_{\rm sd} - V_{\rm Gr}(V_{\rm sd}) \pm \frac{\Delta}{e}\right)}{\epsilon_{\rm MoS}\left(\frac{d_{\rm MoS}}{\epsilon_{\rm MoS}} + \frac{d_{\rm BN}}{\epsilon_{\rm BN}}\right)} = -\frac{\left(V_{\rm sd} - V_{\rm Gr}(V_{\rm sd}) \pm \frac{\Delta}{e}\right)}{d_{\rm MoS}(1+\sigma)}, \qquad \text{(S2b)}$$

$$\mathcal{F}_{\rm BN} = -\frac{\left(V_{\rm sd} - V_{\rm Gr}(V_{\rm sd}) \pm \frac{\Delta}{e}\right)}{\epsilon_{\rm BN}\left(\frac{d_{\rm MoS}}{\epsilon_{\rm MoS}} + \frac{d_{\rm BN}}{\epsilon_{\rm BN}}\right)} = -\frac{\sigma\left(V_{\rm sd} - V_{\rm Gr}(V_{\rm sd}) \pm \frac{\Delta}{e}\right)}{d_{\rm BN}(1+\sigma)}, \qquad \text{(S2c)}$$

with $V_0 = \frac{\epsilon_{\rm MoS}\epsilon_0 \pi(\hbar v)^2}{e^3 d_{\rm MoS}(1+\sigma)}$ and $\sigma = \frac{\epsilon_{\rm MoS}}{\epsilon_{\rm BN}} \frac{d_{\rm BN}}{d_{\rm MoS}}$.

Next, we derive equation (1) in the main text calculating under-barrier action and expanding it up to linear order in electric field and ferroelectric potential drop as follows:

$$S(\varepsilon, V_{sd}) = \int_0^{d_{\rm M}} \sqrt{\frac{2m_{\rm MoS}}{\hbar^2}} \sqrt{\varepsilon_{\pm,MoS}(z) - \varepsilon dz} + \int_{d_M}^{d_{\rm B}+d_M} \sqrt{\frac{2m_{\rm BN}}{\hbar^2}} \sqrt{\varepsilon_{\pm,BN}(z) - \varepsilon dz}$$

$$\approx \kappa_{MoS} d_{MoS} - \frac{1}{2}\frac{\kappa_{MoS} d_{MoS}}{U_{\rm MoS}}\left(\varepsilon - \frac{1}{2} e\mathcal{F}_{\rm MoS}(V_{sd}) d_{\rm MoS} \mp \frac{1}{2}\Delta\right)$$

$$+\kappa_{BN} d_{BN} - \frac{1}{2}\frac{\kappa_{BN} d_{BN}}{U_{\rm BN}}\left(\varepsilon - \frac{1}{2} e\mathcal{F}_{\rm BN}(V_{sd}) d_{\rm BN} - e\mathcal{F}_{\rm MoS}(V_{sd}) d_{\rm MoS} \mp \Delta\right) =$$

$$\kappa_{MoS} d_{MoS} + \kappa_{BN} d_{BN} - \frac{1}{2}\frac{\kappa_{BN} d_{BN}}{|U_{\rm BN}|}\left[(\theta - 1)\left(\varepsilon \mp \frac{\sigma\Delta}{2+2\sigma}\right) + \frac{e\left(V_{sd} - V_{\rm Gr}(V_{\rm sd})\right)}{(2+2\sigma)}(\theta - 2 - \sigma)\right] \qquad \text{(S3)}$$

Here, $\kappa_{MoS} = \sqrt{\frac{2m_{\rm MoS}}{\hbar^2 U_{\rm MoS}}}$, $\kappa_{BN} = \sqrt{\frac{2m_{\rm BN}}{\hbar^2 U_{\rm BN}}}$ are inverse decay lengths of electron wave functions in MoS_2 and hBN layers ($U_{\rm BN} < 0$, $m_{\rm BN} < 0$), respectively, $\theta = \frac{d_{MoS}}{d_{BN}} \sqrt{\frac{m_{\rm MoS} U_{\rm BN}^3}{m_{\rm BN} U_{\rm MoS}^3}} > 0$, and $e > 0$ is the elementary charge. In equation (S3) we used expressions (S2(b-c)) for electric fields in MoS_2 and hBN layers.

Then, integrating over energies in equation (1) of the main text we obtain

$$I \propto \int_{0}^{-eV_{\rm sd}} e^{-2S(\varepsilon)} d\varepsilon =$$

$$= \frac{e^{-2(\kappa_{MoS} d_{MoS} + \kappa_{BN} d_{BN})}}{\frac{\kappa_{MoS} d_{MoS}}{U_{\rm MoS}} + \frac{\kappa_{BN} d_{BN}}{U_{\rm BN}}} \times$$

$$\times \left(\mathrm{Exp}\left\{ \frac{\kappa_{BN} d_{BN} (\theta-\sigma+2\sigma\theta)}{|U_{\rm BN}|(2+2\sigma)} \left[-eV_{\rm sd} - eV_{\rm Gr}(V_{\rm sd}) \frac{(\theta-\sigma-2)}{(\theta-\sigma+2\sigma\theta)} \mp \frac{\sigma(\theta-1)}{(\theta-\sigma+2\sigma\ )} \Delta \right] \right\} - \mathrm{Exp}\left\{ \frac{\kappa_{BN} d_{BN} (\theta-\sigma-2)}{|U_{\rm BN}|(2+2\sigma)} \left[e\left(V_{sd} - V_{\rm Gr}(V_{\rm sd})\right) \mp \frac{\sigma(\theta-1)}{(\theta-\sigma-2)} \Delta \right] \right\} \right) \quad \text{(S4).}$$

Bias voltage dependence of dI/dV$_{sd}$, based on Eq. (S4), is shown in Fig. S5 for domains with –Δ and + Δ polarisations. Solid lines demonstrate the dependence with account of graphene quantum capacitance of graphene (i.e. $V_{\rm Gr} \neq 0$ in Eq. (S4)), whereas dashed lines are for the case when it is infinite (i.e. $V_{\rm Gr} = 0$). One can see that account of graphene quantum capacitance produces only minor quantitative changes, and does not allow one to introduce analytical expression for splitting between curves for oppositely polarised domains. That is why in equation (2) of the main text we used Eq. (S4) neglecting quantum capacitance of graphene and introduced

$$\delta_{>} = \frac{2\sigma(\theta-1)}{\theta-\sigma-2} \Delta \approx 231 \text{ meV}, \quad \text{(S5)}$$

$$\delta_{<} = \frac{2\sigma(\theta-1)}{\theta-\sigma+2\theta} \Delta \approx 48 \text{ meV}, \quad \text{(S6)}$$

with numerical estimates obtained at $\sigma \approx 1.68$ and $\theta \approx 32.5$ for $m_{\rm MoS} = -m_{\rm BN} = m_0$ (m_0 is the free electron mass), $U_{\rm BN} = -2.66$ eV, $U_{\rm MoS} = 0.3$ eV, $d_{BN} = 10$ Å, $d_{MoS} = 12.3$ Å, $\epsilon_{\rm BN} = 3$, $\epsilon_{\rm MoS} = 6.2$.

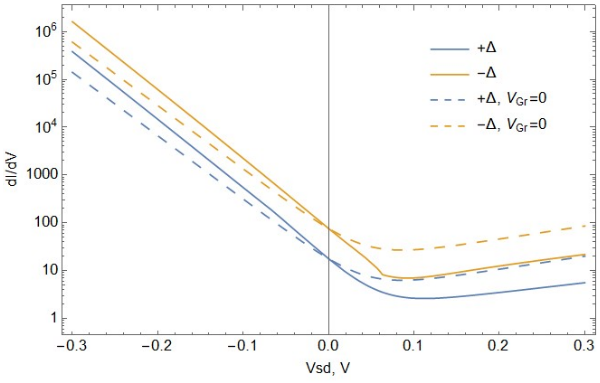

Supplementary Figure S8: Theoretical dI/dV$_{sd}$ -dependence obtained from Eq. (S4). Solid lines show dI/dV vs V$_{sd}$ for the case when graphene quantum capacitance has been taken into account, whereas dashed lines show the same but with infinite quantum capacitance of graphene (i.e. graphene potential is zero, V$_{Gr}$=0).

6. Additional Theoretical modelling

One may worry about an instability of a homogeneous out-of-plane polarisation of a 2D film, due to the dipole-dipole interaction. This can be regarded as a non-local reduction of the energy cost of a domain wall, as the interaction between dipole moments in equally polarised domains leads to a higher energy as compared to their antiparallel orientation. To assess the relevance of such contribution, we compare dipole-dipole interaction energies of two halves of a large area of the MoS_2/MoS_2 interface with out-of-plane dipole moment density P_z for x>0 and $\pm P_z$ for x<0, calculated per unit length of the domain wall:

$$W = \pm \frac{P_z^2}{4\pi\epsilon_{\rm BN}\epsilon_0}\int_{-L}^{-\delta} dx_1 \int_{\delta}^{L} dx_2 \int_{-\infty}^{\infty} dy \, \frac{1}{[\,(x_1-x_2)^2+y^2]^{3/2}} = \pm \frac{P_z^2}{2\pi\epsilon_{\rm BN}\epsilon_0}\ln\left[\frac{L}{4\delta}\right]. \qquad \text{(S7)}$$

Here, $'+'$ corresponds to a mono-domain plane and '-' to two opposite polarised domains separated by a domain wall, which energy is reduced by

$$\Delta W \approx -\frac{P_z^2}{\pi\epsilon_{\rm BN}\epsilon_0}\ln\left(\frac{L}{4\delta}\right) \sim -\,6\,\frac{\mu eV}{nm}\ln\left(\frac{L}{4\delta}\right). \qquad \text{(S8)}$$

In these expressions, a cut-off δ is of the order of TMD's crystal lattice constant, and L is the lateral sample size or the distance to the metallic gate, and $\epsilon_{\rm BN}\sim 3$ is dielectric constant of encapsulation environment. The effect of ΔW should be compared to the deformation energy density of domain wall, $\sim$ 1 eV/nm, hence, can be neglected for all experimentally relevant sizes of flakes, despite a logarithmic factor in it.

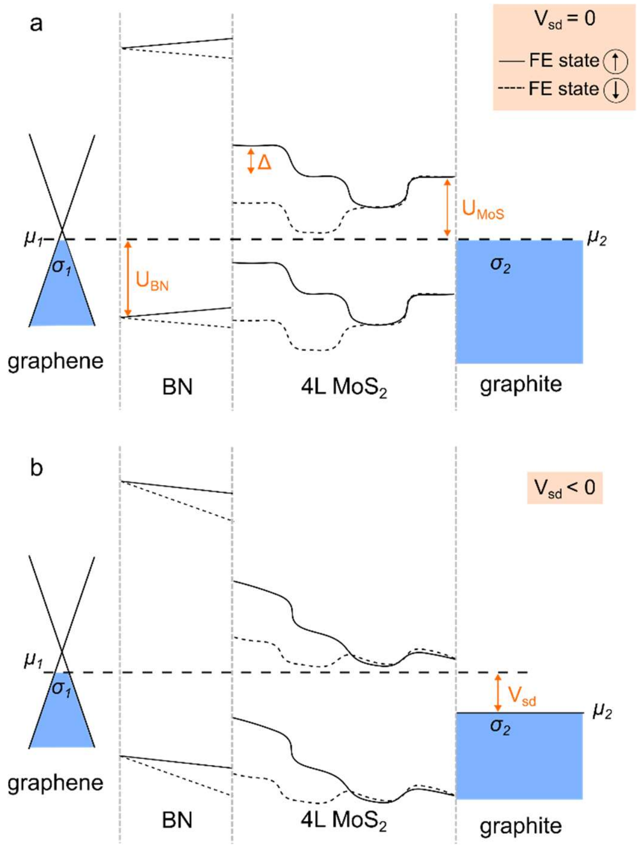

Supplementary Figure S9: **Tunnelling junction for a MoS_2 twisted double bilayer.** (a) Schematic band diagram with equipotential source and drain, V_{sd}=0, and (b) applying a small reverse bias V_{sd}. In the double bilayer there are two additional Δ steps due to the two extra layer interfaces compared to the MoS_2 bilayer.

A possible path towards further increase of ON current ratio for the two FE polarisations states could be by using twisted double bilayer systems with thinner hBN barriers. Those could be made of 2H bilayers with parallel-oriented TMD's unit cells at the interface, of R bilayers (sourced from bulk 3R crystals, as illustrated in Fig. S9).

Supplementary References

1. Kim, K. *et al.* Van der Waals Heterostructures with High Accuracy Rotational Alignment. *Nano Lett.* **16**, 1989–1995 (2016).

2. Weston, A. *et al.* Atomic reconstruction in twisted bilayers of transition metal dichalcogenides. *Nat. Nanotechnol.* **15**, 592–597 (2020).

3. Magorrian, S. J. *et al.* Band alignment and interlayer hybridisation in transition metal dichalcogenide/hexagonal boron nitride heterostructures. *2D Mater.* **9**, (2022).

4. Ogawa, S. *et al.* Band alignment determination of bulk h-BN and graphene/h-BN laminates using photoelectron emission microscopy. *J. Appl. Phys.* **125**, (2019).

5. Miwa, J. A. *et al.* Van der Waals Epitaxy of Two-Dimensional MoS2-Graphene Heterostructures in Ultrahigh Vacuum. *ACS Nano* **9**, 6502–6510 (2015).

6. Pierucci, D. *et al.* Band alignment and minigaps in monolayer MoS2-graphene van der Waals heterostructures. *Nano Lett.* **16**, 4054–4061 (2016).

7. Pierucci, D. *et al.* Large area molybdenum disulphide- epitaxial graphene vertical Van der Waals heterostructures. *Sci. Rep.* **6**, 1–10 (2016).

8. Schuster, R., Habenicht, C., Ahmad, M., Knupfer, M. & Büchner, B. Direct observation of the lowest indirect exciton state in the bulk of hexagonal boron nitride. *Phys. Rev. B* **97**, 041201 (2018).

9. Cassabois, G., Valvin, P. & Gil, B. Hexagonal boron nitride is an indirect bandgap semiconductor. *Nat. Photonics* **10**, 262–266 (2016).

10. Mak, K. F., Lee, C., Hone, J., Shan, J. & Heinz, T. F. Atomically thin MoS2: A new direct-gap semiconductor. *Phys. Rev. Lett.* **105**, 136805 (2010).

11. Trainer, D. J. *et al.* Inter-Layer Coupling Induced Valence Band Edge Shift in Mono- to Few-Layer MoS 2. *Sci. Rep.* **7**, (2017).

12. Chiodini, S. *et al.* Moiré Modulation of Van der Waals Potential in Twisted Hexagonal Boron Nitride. *ACS Nano* (2021) doi:10.1021/acsnano.1c11107.

13. Nečas, D. & Klapetek, P. Gwyddion: An open-source software for SPM data analysis. *Cent. Eur. J. Phys.* **10**, 181–188 (2012).
